# Cross-Species Rhesus Cytomegalovirus Infection of Cynomolgus Macaques

**DOI:** 10.1371/journal.ppat.1006014

**Published:** 2016-11-09

**Authors:** Benjamin J. Burwitz, Daniel Malouli, Benjamin N. Bimber, Jason S. Reed, Abigail B. Ventura, Meaghan H. Hancock, Luke S. Uebelhoer, Amruta Bhusari, Katherine B. Hammond, Renee G. Espinosa Trethewy, Alex Klug, Alfred W. Legasse, Michael K. Axthelm, Jay A. Nelson, Byung S. Park, Daniel N. Streblow, Scott G. Hansen, Louis J. Picker, Klaus Früh, Jonah B. Sacha

**Affiliations:** 1 Vaccine and Gene Therapy Institute, Oregon Health and Science University, Beaverton, Oregon, United States of America; 2 Oregon National Primate Research Center, Oregon Health and Science University, Beaverton, Oregon, United States of America; Blumburg Institute, UNITED STATES

## Abstract

Cytomegaloviruses (CMV) are highly species-specific due to millennia of co-evolution and adaptation to their host, with no successful experimental cross-species infection in primates reported to date. Accordingly, full genome phylogenetic analysis of multiple new CMV field isolates derived from two closely related nonhuman primate species, Indian-origin rhesus macaques (RM) and Mauritian-origin cynomolgus macaques (MCM), revealed distinct and tight lineage clustering according to the species of origin, with MCM CMV isolates mirroring the limited genetic diversity of their primate host that underwent a population bottleneck 400 years ago. Despite the ability of Rhesus CMV (RhCMV) laboratory strain 68–1 to replicate efficiently in MCM fibroblasts and potently inhibit antigen presentation to MCM T cells *in vitro*, RhCMV 68–1 failed to productively infect MCM *in vivo*, even in the absence of host CD8+ T and NK cells. In contrast, RhCMV clone 68–1.2, genetically repaired to express the homologues of the HCMV anti-apoptosis gene UL36 and epithelial cell tropism genes UL128 and UL130 absent in 68–1, efficiently infected MCM as evidenced by the induction of transgene-specific T cells and virus shedding. Recombinant variants of RhCMV 68–1 and 68–1.2 revealed that expression of either UL36 or UL128 together with UL130 enabled productive MCM infection, indicating that multiple layers of cross-species restriction operate even between closely related hosts. Cumulatively, these results implicate cell tropism and evasion of apoptosis as critical determinants of CMV transmission across primate species barriers, and extend the macaque model of human CMV infection and immunology to MCM, a nonhuman primate species with uniquely simplified host immunogenetics.

## Author Summary

Nonhuman primates are excellent models of HCMV infection and immunity, and their CMVs closely resemble HCMV in genomic organization and coding potential. Here, we expand the existing models by isolating new full-length, non-human primate CMV strains that more closely resemble primary HCMV isolates. In addition, we identify determinants needed for recombinant clones of RhCMV to infect MCM both *in vitro* and *in vivo*. Given their simplified immunogenetics, these results enable the use of MCM to deepen our understanding of CMV immunology.

## Introduction

Cytomegaloviruses (CMV) are large, double-stranded DNA viruses of the family *herpesviridae* that induce life-long infections. They are found across a broad range of species, and have adapted to their respective hosts over millions of years of co-evolution. For this reason, CMVs are highly species-specific *in vivo*, with no experimental cross-species transmissions reported in the literature, although anecdotal evidence of accidental cross-species infections exist [1–4]. Some CMV species can replicate to a certain extent in cells of closely related species *in vitro* [5], but replication in cells of more distant species is generally prohibited due to the inability of the virus to block apoptosis or innate defense pathways [6,7].

CMVs of multiple nonhuman primate species including rhesus macaques (RM) and cynomolgus macaques (CM) have been described [8,9], opening up invaluable surrogate models for the study of HCMV virulence, pathogenesis, and immunogenicity, as well as CMV-based vaccine development. The rhesus macaque CMV (RhCMV) genome has been fully sequenced and annotated [10]. A comparison between the coding potential of RhCMV and HCMV genomes revealed a higher degree of conservation of viral ORFs and gene families between the two species than previously estimated [11], far exceeding the homology between MCMV and HCMV. In addition, RhCMV has been cloned as a bacterial artificial chromosome (BAC), allowing for genomic manipulation [12]. We have utilized this BAC technology to design RhCMV vectors expressing SIV antigens, and RM vaccinated with these vectors show unprecedented protection against highly virulent SIVmac239 challenge [13,14]. This protection is associated with unconventional CD8+ T cell responses that are either MHC-II or MHC-E-restricted [15,16]. These unconventional CD8+ T cell responses may be the result of the unique MHC complexity present in RM [17], or the result of conserved immunoregulatory mechanisms utilized by CMV. In order to parse out the importance of host immunogenetics from strain-specific CMV mechanisms, additional nonhuman primate models of CMV infection are needed.

CM are a newly emerging species for the study of CMV. The first two sequences of cynomolgus macaque CMV (CyCMV) were recently published, and contain multiple elongations, truncations, and deletions compared to predicted full-length RhCMV genomes [9,18,19]. Unfortunately, CyCMV has yet to be cloned as a BAC, precluding manipulation of the CyCMV genome for study in CM. Marsh *et*. *al*. recently reported that RhCMV 68–1, the strain used to construct our vaccine vectors, is incapable of infecting Mauritian cynomolgus macaques (MCM), a population with simplified MHC genetics [20–24]. These results cast doubt on CM as a surrogate model for testing of RhCMV vaccine vectors. However, during *in vitro* propagation RhCMV 68–1 lost multiple genes that may confer additional fitness to RhCMV in the setting of cross-species transmission. First, due to an inversion in the ULb’ region that also resulted in the loss of genetic information on either side of the inverted DNA segment, the surface glycoproteins Rh157.5 and Rh157.4 (henceforth referred to by the HCMV homologue names UL128 and UL130, respectively) are deleted [25]. This results in the loss of a functional pentameric complex that mediates non-fibroblast cell tropism [26] as well as shedding and horizontal transmission [27]. Second, the same inversion also results in the deletion of three alpha-chemokine-like open reading frames (ORFs) of the UL146 family encoded by RhCMV, although the functional consequences of this loss have not been elucidated [25]. Third, a premature stop codon in Rh60/Rh61 (henceforth referred to by the HCMV homologue name UL36) has rendered the anti-apoptotic viral inhibitor of caspase-8 activation (vICA) protein nonfunctional [28]. Finally, multiple point mutations have been acquired by the virus during *in vitro* culture prior to BAC cloning which resulted in premature stop codons and frame shifts in at least three ORFs, Rh13.1 (RL13), Rh152./Rh151 (UL119/UL118) and Rh197 (US14D) [11].

**Fig 1:**
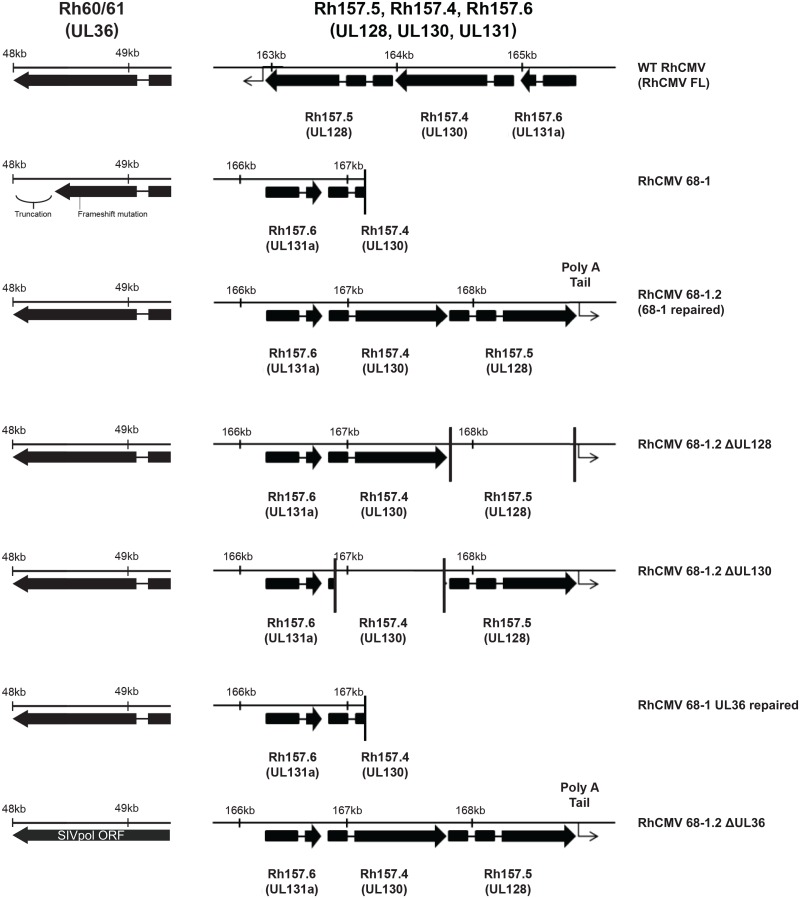
Gene map of laboratory strains of RhCMV. Gene map shows UL36, UL128, and UL130,genes as expressed by different laboratory strains of RhCMV. Each box represents a distinct exon.

Importantly, a partially repaired clone of RhCMV 68–1 has been generated, termed RhCMV 68–1.2, in which expression and functionality of the UL36, UL128, and UL130 gene products have been restored (Table 1, Fig 1) [5]. In addition, we have BAC engineered multiple variant RhCMV clones expressing different combinations of UL36, UL128, and UL130, all of which are based on RhCMV 68–1 or 68–1.2 (Fig 1). Here, we set out to determine if repair of these genes would enable cross-species RhCMV infection of MCM. We show that RhCMV 68–1 and RhCMV 68–1.2 both replicate in cultured primary MCM fibroblasts, supporting previous reports of cross-species CMV infections *in vitro* [5]. However, we did not observe RhCMV 68–1 infection of MCM *in vivo*, supporting the previous findings of Marsh et al [24]. In contrast, RhCMV 68–1.2 established a productive infection as measured by generation of transgene-specific T cell immunity and shedding of virus in urine. We further assessed the roles of UL36, UL128, and UL130 in the cross-species transmission of RhCMV 68–1.2 to MCM, and show that expression of either UL128 together with UL130, or UL36 alone is sufficient for infection. Thus, we show for the first time that experimental, cross-species RhCMV infection of CM is possible. These results pave the way for utilizing RhCMV vaccine vectors in the CM model, and shed light on the mechanisms restricting cross-species transmission of CMV.

**Table 1:** RhCMV vectors based on expression of UL36, UL128, UL130, and the Pentameric Complex (PC).

	HCMV Homologues			
RhCMV strains	UL36	UL128	UL130	PC
RhCMV 68–1	−	−	−	−
RhCMV 68–1.2	+	+	+	+
RhCMV 68–1.2 (ΔUL128)	+	−	+	−
RhCMV 68–1.2 (ΔUL130)	+	+	−	−
RhCMV 68–1 (UL36R)	+	−	−	−
RhCMV 68–1.2 (ΔUL36)	−	+	+	+

## Results

### Isolation and deep sequencing analysis of novel RhCMV and CyCMV strains

**Fig 2:**
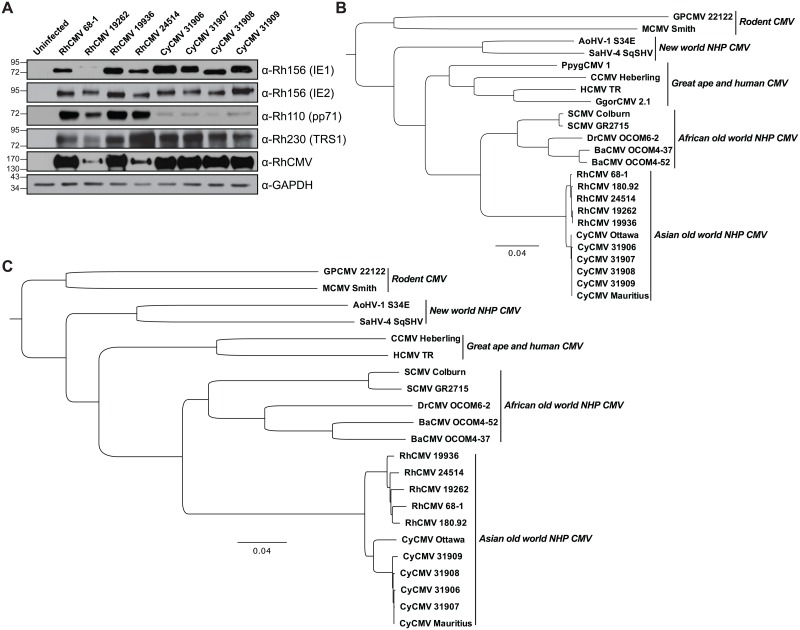
Characterization of primary RhCMV and CyCMV isolates. A) Immunoblotting of cell lysates from primary rhesus fibroblasts infected with RhCMV and CyCMV isolates using RhCMV-specific antibodies. B) Neighbor-joining phylogenetic analysis was performed using full-length amino acid sequence from the highly conserved DNA polymerase UL54 from using rhesus and cynomolgus macaque CMV isolates, along with previously described isolates from human, non-human primates, and rodents. Bootstrap support for each node is indicated. C) Similar phylogenetic analysis was performed using the full-length nucleotide sequence for the same strains as B, except GgorCMV was excluded because the full genome was not available.

Comparative analyses between non-human primate CMV strains are limited due to a paucity of field isolate sequences published to date. To more thoroughly examine the similarities and differences between RhCMV and CyCMV, we collected urine from Indian-origin RM born and housed at the Oregon National Primate Research Center (ONPRC) and MCM that were born and weaned on Mauritius prior to arriving at ONPRC, and isolated CMV from these samples through co-culture on primary rhesus fibroblasts. Co-culturing of urine from three RM (19262, 19936 and 24514) resulted in cytopathic effect (CPE) consistent with RhCMV infection, and cell lysates of primary rhesus fibroblasts showed strong immunoblotting reactivity with RhCMV specific antibodies (Fig 2A). We employed these same techniques to isolate CyCMV from four MCM, using RhCMV antibodies for immunoblotting of infected primary rhesus fibroblast lysates to detect expression of both immediate-early and late CyCMV proteins (Fig 2A).

To acquire full-length CMV isolates with as little *in vitro* adaptation as possible, passaging of the virus was kept to an absolute minimum, with no more than 4 passages of *in vitro* culture prior to sequencing analysis. We then deep sequenced purified viral DNA to further evaluate our new field isolates and obtained full genome sequence coverage for all seven novel CMV isolates. To compare our field isolates to other previously described human, nonhuman primate, and rodent CMV strains [8], we performed phylogenetic analyses using alignments of DNA sequences of the highly conserved DNA polymerase UL54 (Fig 2B), or of the full genome (Fig 2C). While RhCMVs and CyCMVs are closely related to each other, they still clustered separately according to the host species. Our novel RhCMV isolates were grouped with the RhCMV laboratory strains 68–1 and 180.92, whereas all of our MCM CyCMV isolates were closely aligned with the recently published isolate CyCMV Mauritius [19] and demonstrated a close sequence relationship to CyCMV Ottawa, an isolate derived from a Filipino-origin CM [9]. Thus, we have successfully isolated seven novel RhCMV and MCM CyCMV from animals housed at ONPRC.

### Assessment of the coding potential of the novel RhCMV and CyCMV isolates

We next examined the coding potential of our RhCMV and CyCMV genomes to identify any adaptations acquired during *in vitro* passage. Common genomic adaptations have been defined through the study of RhCMV 68–1, which was passaged extensively *in vitro* on fibroblasts prior to being cloned as a BAC [11]. These *in vitro* adaptations were identified by comparing the cloned RhCMV 68–1 BAC sequence with other non-clonal RhCMV sequences, including those acquired directly from the original urine source collected in 1968 [10,29]. Interestingly, similar adaptations such as inactivation of UL36, UL128 or UL130 are frequently observed in HCMV [30–32].

**Fig 3:**
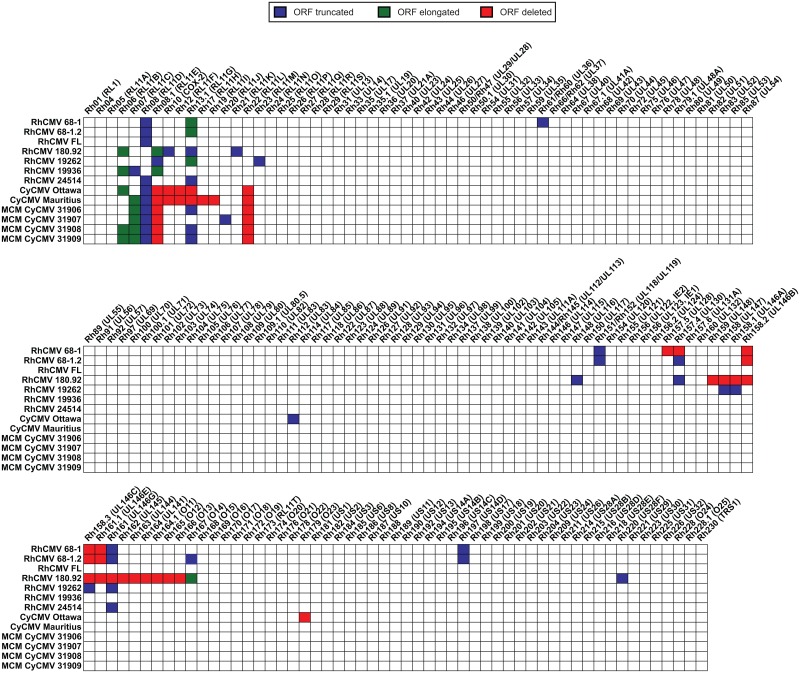
Coding potential of RhCMV and CyCMV strains. Full genomes of our viral isolates were assembled and aligned to each other to compare the predicted viral coding regions. Start and stop codons were determined by comparison of all published RhCMV and CyCMV genomes and the predicted full genome coding content of RhCMV [11] was used to determine maximal low passage viral coding potential. Hypothetical full length strain 68–1 with all lab adaptations repaired was included as a theoretical full length isolate [25,29]. ORFs in our isolates showing frame shift mutations or point mutations leading to the usage of a premature stop codons compared to the majority predicted stop codon leading to shortened ORFs are indicated in blue, whereas ORF containing frameshift leading to elongations compared to the predicted majority sequence are indicated in green. ORFs that are fully or mostly missing in our isolates compared to and the predicted full genome coding content of RhCMV are highlighted in red.

To identify the most probable start and stop codons for all annotated ORFs, we generated a consensus genome sequence across all RhCMV and CyCMV strains and then compared each strain against this consensus sequence. This enabled us to determine if any viral sequence lost genetic information or acquired point mutations leading to frame shifts and premature stop codons in coding regions. A hypothetical full length RhCMV (FL), derived by combining the sequence information of RhCMV 68–1 BAC with primary RhCMV isolate sequences, contains the entire ULb' region and encodes complete versions of the Rh13.1 (RL13), Rh61/Rh60 (UL36), Rh152/Rh151 (UL119/UL118) and Rh197 (US14D) ORFs, all of which are truncated, elongated, or deleted in RhCMV strain 68–1 (Fig 3). In contrast, RhCMV strain 180.92 lacks a genome fragment ranging from Rh159 through Rh166 in addition to frame shift mutations in the Rh06 (RL11B), Rh08.1 (RL11E), Rh10 (vCOX-2), Rh13.1 (RL13, RL11G), Rh21 (RL11K), Rh148 (UL116), Rh157.4 (UL130), Rh167 (O14), and Rh220 (US28F) ORFs leading to either shortened or elongated coding regions (Fig 3). Compared to RhCMV 68–1 and 180.92, the new RhCMV isolates closely correspond to the predicted full-length sequence. RhCMV isolate 19262 has frame shifts in seven different genes, whereas RhCMV isolates 19936 and 24514 have frame shifts in three different genes (Fig 3, S1 Table).

We next assessed the coding potential of the new CyCMV isolates, again comparing each strain against an overall CMV consensus sequence. The two published CyCMV isolates (Ottawa and Mauritius) have been annotated previously, but unique annotations for each virus make it difficult to compare ORFs across strains and species. Therefore, we re-annotated all CyCMV isolates using the same nomenclature originally introduced for RhCMV [10], but giving the ORFs a Cy prefix to indicate their CyCMV origin. This approach revealed that both genomes of the two published CyCMV isolates, Ottawa and Mauritius, display a multi-kb loss of genetic information likely due to *in vitro* adaptation, with CyCMV Mauritius lacking seven ORFs and CyCMV Ottawa lacking six (Fig 3). Interestingly, both strains have a deletion in a very similar region near the 5’ end of the genome surrounding Cy13.1 (RL13, RL11G). Deletion or mutation of RL13, a known Fc binding protein [33], provides HCMV with a growth advantage *in vitro* [34]. This ORF also appears to be under strong negative selective pressure in macaque CMV, as it is also mutated in RhCMV strains 68–1, 180.92, and in four of our novel isolates (Fig 3). This similarity indicates that Rh13.1, Cy13.1, and RL13 may be mechanistically conserved as we have previously proposed [11]. Interestingly, CyCMVs do not encode ORFs that are homologous to Rh08.1 (RL11E) and Rh22 (RL11L) (Fig 3). The complete genome sequences of primary isolates of RhCMV and CyCMV thus enabled the validation and correction of previous genome annotations.

### Nucleotide diversity in RhCMV and CyCMV low passage isolates

**Fig 4:**
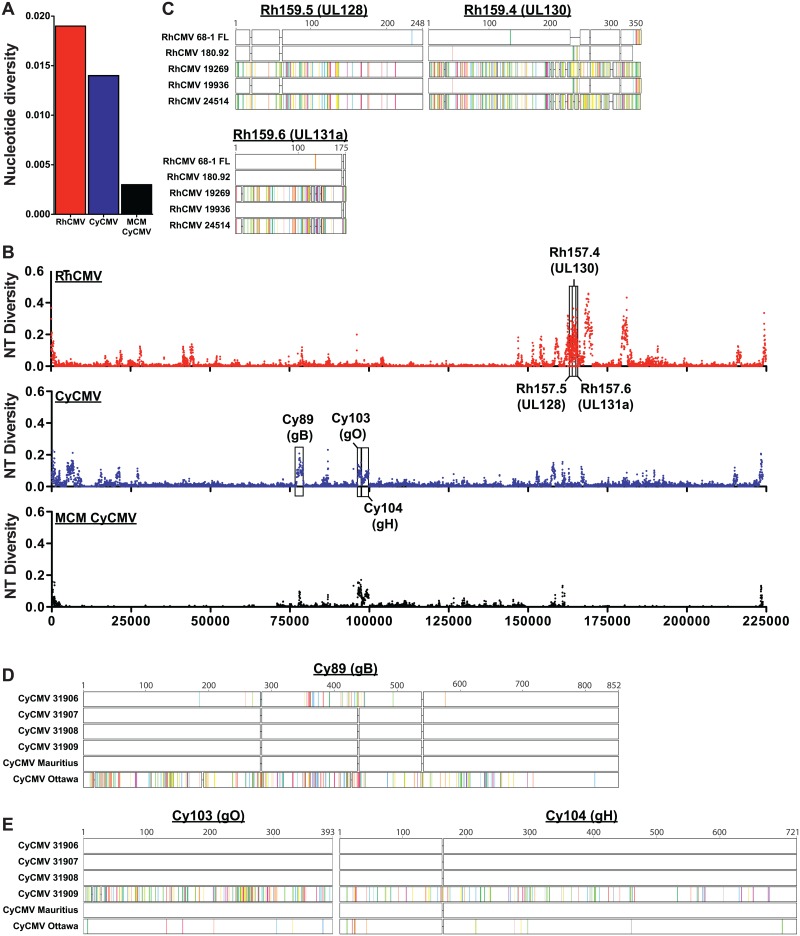
Nucleotide sequence variation across RhCMV and CyCMV. A) Overall nucleotide diversity within either RhCMV isolates, CyCMV isolates, or MCM CyCMV isolates. B) Graphs indicate the nucleotide diversity across the genome, calculated using overlapping 100 bp sliding windows, incrementing by 50 bp per window. C) Amino acid alignments showing changes in UL128, UL130 and UL131a for each strain when compared to the consensus sequence. D) Amino acid alignments showing changes in gB, or E) gO and gH for each strain when compared against the consensus sequence. Colored dashes represent amino acid differences from the CMV consensus sequence.

We next calculated the overall inter-strain nucleotide diversity between our CMV isolates. This measure of polymorphism within a population had a value of 0.019 (19 nucleotide differences per 1000 genomic bases) across all RhCMV genomic sequences (Fig 4A). This nucleotide diversity is slightly lower than the diversity determined for 100 newly isolated geographically diverse HCMV strains (0.026) [35], likely due to our limited sample size of RhCMV and the fact that all viruses were isolated from Indian-origin RM housed at ONPRC. Interestingly, the nucleotide diversity across all MCM CyCMV isolates (including CyCMV Mauritius) was only 0.003 (Fig 4A). It should be noted that the four ONPRC MCMs were imported directly from the island of Mauritius, and should represent active circulating CMV strains from this island. While our sample size of five MCMs lacks the statistical power to completely rule out the presence of additional strains in the entire MCM population, the lower diversity observed in MCM CyCMV compared to RhCMV is in alignment with the natural history of MCM which experienced a severe population bottleneck approximately 400 years ago [36]. Inclusion of CyCMV Ottawa, which was derived from a Filipino-origin CM, increased the level of nucleotide diversity to 0.014 (Fig 4A). This nucleotide diversity is similar to comparisons across RhCMV strains, indicating that inter-strain diversity exists in CyCMV, but that it is largely absent in MCM CyCMV isolates.

We further assessed the nucleotide diversity across the entire genome to elucidate inter-strain differences (Fig 4B). This analysis revealed that RhCMV diversity is not equally distributed across the genome, but localized to distinct genomic regions. A similar analysis of HCMV indicated that most ORFs of the genome are highly conserved across global isolates while the RL11 gene family, chemokines of the UL146 gene family, and specific viral surface glycoproteins exhibit significant diversity [37]. Similarly, the RL11 gene family in RhCMV shows strain diversity, although less extensive than HCMV, and the viral chemokines of the UL146 gene family are the most diverse ORFs in the genome (Fig 4B). Surprisingly however, the pentameric complex members UL128, UL130 and UL131a are diverse in RhCMV whereas they show a very high degree of conservation in HCMV (Fig 4C).

As discussed above, MCM CyCMV strains show very limited sequence diversity. Moreover, this diversity is highly localized to genes Cy89, Cy103 and Cy104, encoding the predicted homologs of HCMV glycoproteins gB, gO, and gH, respectively. CyCMV 31906 encodes for a gB allele that is substantially different from other CyCMVs (Fig 4D), whereas gO and gH in CyCMV 31909 are distinct (Fig 4E). Thus, although the majority of the genomic sequence of MCM CyCMV is highly conserved, the glycoprotein sequences indicate that at least two distinct CyCMV genotypes are present within the MCM population. Similarly, distinct alleles of gB, gO, and gH are used for genotyping of HCMV, with substantially different sequences generally indicating infection by distinct HCMV genotypes [38–40].

### RhCMV infection of primary MCM fibroblasts *in vitro*

**Fig 5:**
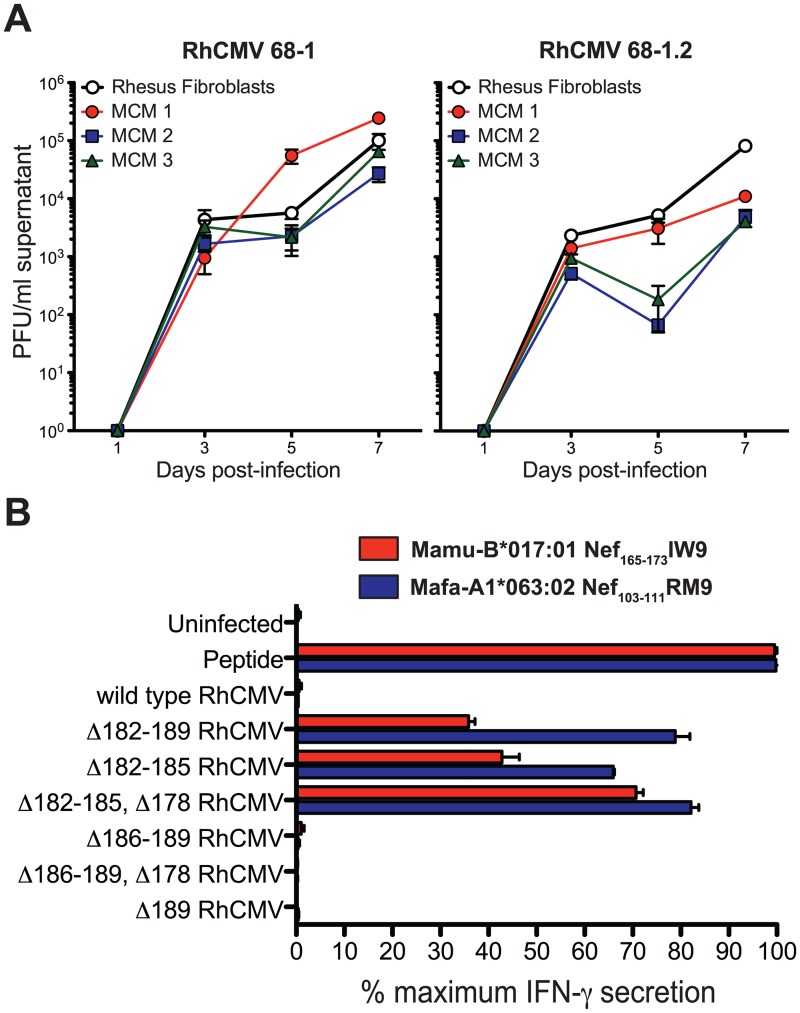
RhCMV replicates on primary MCM fibroblasts *in vitro* and compromises MHC-I presentation of SIV transgene peptides. A) Multi-step growth curves reveal the ability of RhCMV 68–1 and RhCMV 68–1.2 to replicate on primary RM and MCM fibroblasts (PFU = plaque forming units). B) ELISpot shows IFNγ secretion by SIV-specific CD8+ T cell lines derived from RM and MCM following stimulation with RhCMV/SIV vector-infected, MHC-matched primary fibroblasts (compared to IFNγ secretion following stimulation with peptide-pulsed primary fibroblasts).

Given the high homology in gene content between RhCMV and MCM CyCMV, we tested whether RhCMV was capable of infecting primary MCM fibroblasts. We performed multi-step growth curves on primary MCM and RM fibroblasts infected with RhCMV 68–1 and the partially repaired clone 68–1.2 containing intact UL36, UL128 and UL130 homologs [5]. Both RhCMV clones infected primary MCM fibroblasts and showed similar growth kinetics and peak titers (Fig 5A), indicating that the rhesus virus is able to productively infect cynomolgus cells.

Since this finding indicates that RhCMV can overcome cell-intrinsic innate immunity of cynomolgus cells we next examined if RhCMV could also evade adaptive immune recognition in the context of this cross-species infection. CMV encodes multiple immune evasion vectors that powerfully interrupt CD8+ T cell antigen processing and presentation [41]. Indeed, RhCMV and HCMV express four related glycoproteins—Rh182 (US2), Rh184 (US3), Rh185 (US6), and Rh189 (US11)—that act synergistically with very high efficiency to inhibit presentation of MHC class I-restricted epitopes by infected cells [42]. We have previously shown that this MHC class I interference results in the inability of SIVgag-specific CD8+ T cells to recognize fibroblasts infected with RhCMV 68–1 expressing SIVgag [41]. To test whether MHC-I-dependent peptide presentation to CD8+ T cells is similarly altered in RhCMV-infected primary MCM fibroblasts, we measured the ability of an MCM-derived, SIV-specific CD8+ T cell line targeting the Nef_103-111_RM9 epitope to recognize primary MCM fibroblasts infected with a panel of RhCMV 68–1 clones expressing the SIV Rev-Tat-Nef fusion protein (RhCMV/rtn) (Fig 5B, blue). We compared these results to the ability of a RM-derived, SIVnef-specific CD8+ T cell line to recognize primary RM fibroblasts infected with the same panel of RhCMV 68–1 clones (Fig 5B, red). Similar to the previously described inability of SIV-specific T cells to recognize primary RM fibroblasts infected with RhCMV 68–1 expressing SIV antigen [15], the MCM SIVnef-specific CD8+ T cell line did not respond to primary MCM fibroblasts infected with RhCMV/rtn 68–1 containing an intact Rh182-189 region. In contrast, both the RM and MCM SIVnef-specific CD8+ T cell lines recognized multiple RhCMV/rtn 68–1 mutants that had partial or complete deletions of Rh182-185. Thus, RhCMV inhibits T cell antigen presentation in both RM and MCM primary fibroblasts. This evasion mechanism of RhCMV is known to promote superinfection [41], implying that RhCMV should be able to infect CyCMV seropositive MCM.

### RhCMV infection of MCM *in vivo*

**Fig 6:**
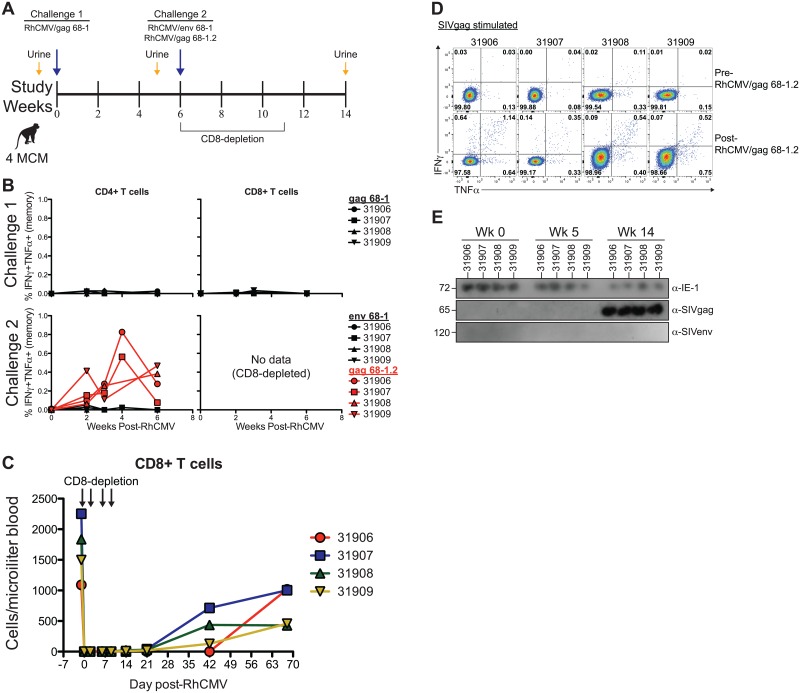
RhCMV 68–1.2 infects MCM. A) Study timeline showing RhCMV challenges and urine collecting in MCM. B) *Ex vivo* T cell responses in blood following RhCMV challenge. Measured as the frequency of IFNγ+TNFα+ T cells following stimulation with peptides spanning SIV protein ORFs. Corrected for frequency within memory T cell compartment. C) Absolute CD8+ cell counts in peripheral blood from four MCM following administration of CD8-depleting antibody. D) *Ex vivo* CD4+ T cell responses in BAL following RhCMV challenge. E) Western blots show shedding of RhCMV/SIV 68–1.2 vectors, but not RhCMV/SIV 68–1 vectors, in the urine of immunocompromised MCM.

Since RhCMV has a similar gene content to MCM CyCMV, and since RhCMV is capable of infecting and inhibiting CD8+ T cell antigen presentation in primary MCM fibroblasts *in vitro* we assessed whether RhCMV is capable of infecting MCM by monitoring SIV-specific T cell response longitudinally in MCM inoculated with various RhCMV recombinants expressing SIV antigens as transgenes (Fig 6A). However, upon subcutaneous (s.c.) inoculation of four MCM with 1 x 10^7^ PFU of 68–1 RhCMV/gag we did not observe CD4+ or CD8+ T cells responding to overlapping peptide pools covering the SIVgag protein in any of the animals during 6 weeks post-challenge. Since the induction of T cell responses is a highly sensitive indicator of viral infection, this result indicates that RhCMV 68–1 was unable to infect MCM (Fig 6B, top row). This result is consistent with previously published results, which showed lack of immune responses, viral shedding, and viral replication upon inoculation of MCM with RhCMV 68–1 [24].

Given this result, we formed two distinct hypotheses to explain the failure of RhCMV 68–1 to infect MCM. First, all four MCM were previously infected with MCM CyCMV (Fig 2A) and we hypothesized that, unlike immunity of RM to RhCMV, immunity against CyCMV could provide protection against RhCMV infection. Second, RhCMV 68–1 has lost the structural genes UL128 and UL130 during passage *in vitro*, and surface expression of these genes is necessary for formation of the pentameric complex, which mediates viral entry into endothelial and epithelial cells (Fig 1) [43]. In addition, RhCMV 68–1 contains an early truncation in the UL36 ORF that renders the anti-apoptotic properties of this protein nonfunctional. Therefore, we hypothesized that, while lack of these genes still permits superinfection of RM, the limited cellular tropism and reduced anti-apoptosis capabilities of RhCMV 68–1 prevented infection of MCM.

To test our hypotheses, we depleted CD8+ cells (both CD8+ T and NK cells in MCM) in the same four MCM prior to s.c. challenge with 1 x 10^7^ PFU of 68–1 RhCMV/env and 68–1.2 RhCMV/gag (Fig 6A and 6C). We then monitored the CD4+ T cell responses against both SIVenv and SIVgag for 6 weeks post-inoculation. Interestingly, inoculation with RhCMV 68-1/env again failed to induce CD4+ T cell responses despite depletion of CD8+ cells (Fig 6B; bottom row). In contrast, RhCMV/gag 68–1.2 induced robust SIVgag-specific CD4+ T cell responses that were maintained through week 6. This finding ruled out that CyCMV-specific CD8+ T cell immunity protected against infection with RhCMV, and instead indicated that UL36, UL128 or UL130, genes missing in RhCMV 68–1, but repaired in RhCMV 68–1.2, are required for cross-species infection of MCM with RhCMV (Fig 1).

Since RhCMV elicits particularly high frequency T cell responses in the lungs of RM we next assessed SIV-specific CD4+ T cell responses in bronchoalveolar lavages (BAL). We detected SIVgag-specific CD4+ T cell responses in the BAL of all four animals inoculated with 68–1.2 RhCMV/gag, but SIVenv-specific CD4+ T cell were below detection limits in MCM inoculated with 68–1 RhCMV/env (Fig 6D). This result shows that T cell immunity at peripheral sites in RhCMV/SIV infected MCM is similar to that found in RM and confirms the absence of RhCMV 68-1-induced immunity.

Finally, we assessed shedding of RhCMV in MCM urine (Fig 6A). When urine from various time points was co-cultured on RM fibroblasts followed by immunoblotting of infected cell lysates we detected CyCMV at all time points using anti-IE antibodies. However, SIVenv expression was not observed at any of the time points indicating lack of 68–1 RhCMV/env shedding consistent with the lack of SIVenv-specific T cell responses. In contrast, SIVgag was detected in cell lysates that were co-cultured with urine from all four animals inoculated with 68–1.2 RhCMV/gag at 14 weeks post-infection (Fig 6E). These data provide definitive evidence that RhCMV 68–1.2 was able to replicate in the MCM host.

### RhCMV 68–1.2 infects immunocompetent MCM

**Fig 7:**
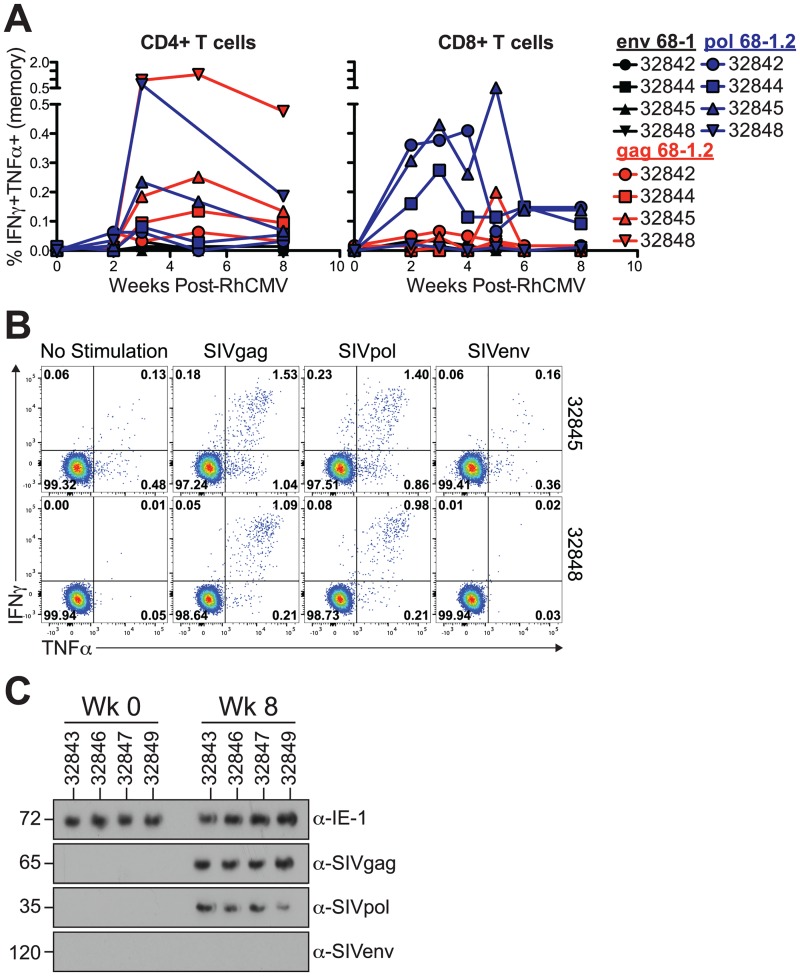
RhCMV 68–1.2 infects immunocompetent MCM. A) *Ex vivo* T cell responses in blood following RhCMV challenge. Measured as the frequency of IFNγ+TNFα+ T cells following stimulation with peptides spanning SIV protein ORFs. Corrected for frequency within memory T cell compartment. B) *Ex vivo* CD4+ T cell responses in BAL following RhCMV challenge. C) Western blots show shedding of RhCMV/SIV 68–1.2 vectors, but not RhCMV/SIV 68–1 vectors, in the urine of fully immunocompetent MCM.

We next sought to determine whether RhCMV 68–1.2 is able to infect fully immunocompetent MCM. To this end, we inoculated a new cohort of four MCM with 68–1 RhCMV/env (1 x 10^7^ PFU), 68–1.2 RhCMV/pol (1 x 10^7^ PFU), and 68–1.2 RhCMV/gag at a reduced dose (2.5 x 10^6^ PFU) without prior CD8+ cell depletion. We again observed CD4+ T cell responses to SIVgag and SIVpol expressed by RhCMV 68–1.2, but not to SIVenv expressed by RhCMV 68–1 (Fig 7A). Similarly, all animals developed SIV-specific CD8+ T cell responses against SIV transgenes expressed by 68–1.2 (Fig 7A). In accordance with the peripheral blood T cells, we identified large CD4+ T cell responses against SIVgag or SIVpol in the BAL of 68–1.2-inoculated animals, but not to SIVenv expressed by 68–1 (Fig 7B). Furthermore, we observed shedding of both RhCMV 68–1.2 vectors, but not 68–1 RhCMV/env in the urine from each of the animals (Fig 7C). Taken together, these results demonstrate that RhCMV 68–1.2 is capable of replicating in fully immunocompetent MCM.

### Formation of the pentameric complex is not required for cross-species RhCMV transmission in the presence of UL36 expression

Compared to RhCMV 68–1, RhCMV 68–1.2 contains the genes UL128 and UL130 derived from RhCMV strain 180.92 (Fig 1, Table 1). UL128 and UL130 encode subunits of the pentameric complex that governs CMV tropism. In addition, UL128 and UL130 show sequence similarities to CCL and CXCL chemokines, respectively, and chemokine activity has been shown for HCMV UL128 *in vitro* [44]. To determine whether infection of MCM required a functional pentameric complex for RhCMV to overcome species restriction we inoculated four MCM with 68–1 RhCMV/env (1 x 10^7^ PFU) and two of these MCM were co-inoculated with either 68–1.2 RhCMVΔUL128/gag (2.5 x 10^6^ PFU) or 68–1.2 RhCMVΔUL130gag (2.5 x 10^6^ PFU).

**Fig 8:**
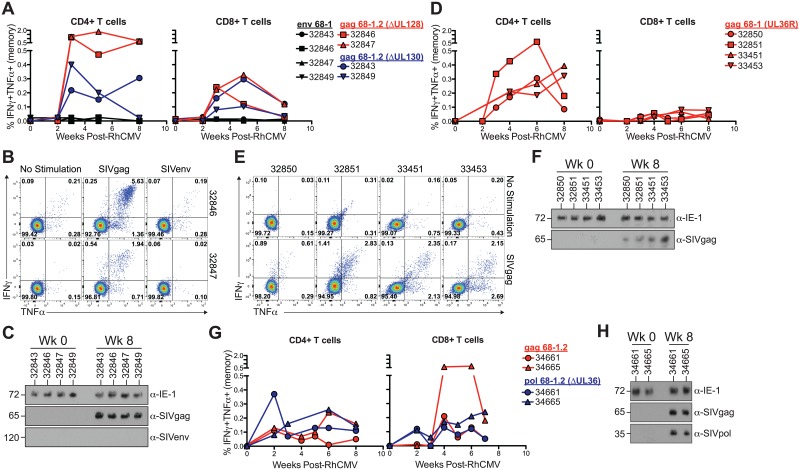
Formation of the pentameric complex is not required for cross-species RhCMV infection. *Ex vivo* T cell responses in blood following RhCMV challenge with either A) 68–1.2 RhCMVΔUL128/gag or 68–1.2 RhCMVΔUL130/gag and 68–1 RhCMV/env, or (D) 68–1 RhCMV/gag (UL36R), or (G) 68–1.2 RhCMV/gag and 68–1.2 RhCMVΔUL36/pol. Measured as the frequency of IFNγ+TNFα+ T cells following stimulation with peptides spanning SIV protein ORFs. Corrected for frequency within memory T cell compartment. (B,E) *Ex vivo* CD4+ T cell responses in BAL following either (B) 68–1.2 RhCMVΔUL128/gag and 68–1 RhCMV/env innoculation, or (E) 68–1 RhCMV/gag (UL36R). (C,F,H) Western blots show shedding of RhCMV/SIV vectors in the urine of MCM following inoculation with C) 68–1.2 RhCMVΔUL128/gag or 68–1.2 RhCMVΔUL130/gag, and 68–1 RhCMV/env, or (F) 68–1 RhCMV/gag (UL36R), or (H) 68–1.2 RhCMV/gag and 68–1.2 RhCMVΔUL36/pol.

We observed robust CD4+ and CD8+ T cell responses to SIVgag in the peripheral blood and BAL of all four MCM, regardless of UL128 or UL130 deletion (Fig 8A and 8B). We also observed shedding in the urine of animals inoculated with ΔUL128 and ΔUL130 versions of 68–1.2 RhCMV/gag (Fig 8C). In contrast, CD4+ or CD8+ T cell responses against SIVenv were not observed, and 68–1 RhCMV/env was not shed in the urine (Fig 8A and 8C). These results indicated that repair of the pentameric complex in 68–1.2 was not the primary factor supporting cross-species infection.

### The anti-apoptotic protein UL36 allows for cross-species RhCMV infection

In addition to the pentameric complex, a disabling mutation in the gene encoding the anti-apoptotic protein UL36 (vICA) was repaired in RhCMV 68–1.2. We therefore also evaluated whether repair of UL36 alone would enable RhCMV 68–1 to infect MCM. We generated RhCMV/gag (UL36R), a recombinant RhCMV 68–1 with a repaired UL36 gene to investigate the role of this protein in RhCMV infection (Fig 1). In support of our data with RhCMV 68–1.2 recombinants expressing intact UL36, but lacking a pentameric complex, we observed robust CD4+ T cell responses against SIVgag in both the blood and BAL of animals after inoculation with 1 x 10^7^ PFU of 68–1 RhCMV/gag (UL36R) (Fig 8D and 8E). Interestingly, although SIVgag-specific CD8+ T cell responses were also observed in the blood, they seemed at a lower frequency (Fig 8D). However, given the small sample size this was not significantly different from 68–1.2-inoculated animals. Importantly, we also observed RhCMV/gag 68–1 (UL36R) shedding in the urine of all four MCM (Fig 8F). Although RhCMV/gag 68–1 (UL36R) lacks the UL128/UL130-containing pentameric complex, RhCMV 68–1 retains the ability to productively infect primary macaque kidney epithelial cells *in vitro* [45]. Given that kidney epithelial cells are a major reservoir for CMV shedding into the urine, this explains how pentamer deficient RhCMV is found in the urine. Indeed, previous studies have demonstrated that the pentamer-deficient RhCMV strain 68–1 is shed into the urine of rhesus macaques *in vivo* [41]. Thus, expression of the anti-apoptotic protein UL36 is sufficient to allow RhCMV 68–1 to establish infection in MCM.

### Expression of the pentameric complex is sufficient for cross-species infection of MCM with RhCMV 68–1.2 in the absence of UL36 expression

Infection with 68–1 RhCMVUL36R/gag suggested that UL36 expression was sufficient for cross-species infection of RhCMV 68–1. To determine whether UL36 was also required for cross-species infection of RhCMV 68–1.2 we deleted UL36 from RhCMV 68–1.2 generating recombinant 68–1.2 RhCMVΔUL36/pol (Fig 1). In this recombinant we replaced UL36 with SIVpol thus ensuring that any SIVpol-specific T cell responses were generated from UL36-deficient vectors. We inoculated two MCM with 1 x 10^7^ PFU each of 68–1.2 RhCMV/gag and 68–1.2 RhCMVΔUL36/pol. Surprisingly, we detected CD4+ and CD8+ T cell responses to both SIVgag and SIVpol, indicating that, in the presence of an intact pentameric complex, UL36 is dispensable for infection of MCM by RhCMV (Fig 8G). This conclusion was further supported by the observation that both 68–1.2 RhCMV/gag and 68–1.2 RhCMVΔUL36/pol were shed in the urine of both animals (Fig 8H). These data indicate that both pentameric complex components and anti-apoptotic proteins independently enable the infection of MCM by RhCMV 68–1 suggesting that species restriction involves multiple layers that each provide a partial, but incomplete barrier to cross-species infection of non-human primate CMVs.

## Discussion

Nonhuman primates are important models of HCMV infection [8]. Here, we have increased our understanding of RhCMV and CyCMV by isolating and sequencing three new RhCMV strains derived from Indian-origin rhesus macaques born and housed at the ONPRC, and four CyCMV isolates derived from cynomolgus macaques originally born on the island of Mauritius. Only two CyCMV strains had been described prior to our present analysis, one from a Filipino-origin and the other from a Mauritius-origin CM [9,19]. Unfortunately, neither strain was full-length and both carried deletions of their RL13 homologue and adjoining regions of the genome. In contrast, all four of our MCM CyCMV genomes contain all predicted ORFs, assuming CyCMVs do not express Rh08.1 (RL11E) and Rh22 (RL11L), an assumption supported by the fact that neither of the previously published CyCMV strains nor any of the four new CyCMV strains published here contain the genetic information for these genes. We also describe the first full-length RhCMV isolate (19936) that contains all previously predicted ORFs and contains no frame shift mutations outside of the highly variable RL11 gene family [11].

Using our expanded genetic data set, we show that MCM CyCMV field isolates exhibit minimal diversity, in accordance with the sharp genetic bottleneck that occurred in this population approximately 400 years ago [36]. The CyCMV genome has collinear genomic organization and almost identical coding content to RhCMV, but full genome phylogenetic analysis shows distinct and tight clustering of CMV strains between the two species. In addition, the nucleotide diversity across RhCMV and CyCMV genomes is similar if all CyCMV from diverse backgrounds are considered, and also the areas of diversity are similar, indicating that both viral species are closely related. This relationship is mirrored on the host side where the two macaque species are so closely connected, that they naturally interbreed in regions where they co-habitate, producing fertile offspring [46–48]. Thus, our genetic data bridge an important gap in our understanding of similarities and differences between RhCMV and CyCMV strains, and support the use of both RM and CM in future research.

Compared to the first complete RhCMV genome (19936) containing all previously predicted ORFs reported here, RhCMV 68–1 lacks multiple genes and contains multiple point mutations consistent with previous comparisons to a hypothetical complete genome [11]. Despite these defects and in contrast to infection in CM, RhCMV 68–1 is fully capable of establishing and maintaining an infection in RM either naturally infected with RhCMV or experimentally infected with RhCMV 68-1-derived recombinant viruses [49]. However, viremia, shedding, and transmission are clearly reduced for RhCMV 68–1 in RM even during primary infection as compared to low passage isolates of RhCMV, indicating that these mutations are attenuating *in vivo* [27]. Presently it is not known which mutations need to be repaired to restore the full replication potential of RhCMV 68–1 in RM, but UL128 and UL130 as well as UL36 studied here in the context of MCM infection are likely candidate genes. We demonstrate that lack of CM-infection by RhCMV 68–1 can be overcome by repair of either UL128 together with UL130, or UL36. These results suggest that repairing one of the two gene regions renders species restriction due to lack of the other gene region less stringent. A possible explanation for this surprising finding comes from the fact that UL36 and UL128/UL130 perform very different functions in the viral life cycle.

UL36 of HCMV and RhCMV, as well as the MCMV homolog M36, all have been shown to inhibit extrinsic apoptosis by impeding localization of FAS-associated protein with a death domain (FADD) to the death-inducing signaling complex (DISC) following TNFR1 or CD95/Fas signaling and thus caspase 8 induction [50,51]. Indeed, a dominant negative FADD can functionally replace M36 and restore full pathogenicity *in vivo* [52]. In the absence of UL36 or M36, CMV-infected cells become more sensitive to FASL or TNF-mediated apoptosis but there is no cell-intrinsic defect [53]. For instance, M36-deficient viruses do not show a growth defect in fibroblasts except in the presence of TNF-producing macrophages [53]. It is conceivable that UL36-deficient RhCMV renders CM cells more sensitive to extrinsic apoptosis signals than RM cells potentially due to non-UL36-mediated, anti-apoptotic mechanisms that can partially compensate for the loss of UL36 in RM, but not in CM. A potential candidate is UL37 (viral mitochondrial inhibitor of apoptosis; vMIA), which binds the pro-apoptotic, mitochondrial-associated protein Bax, preventing mitochondrial permeabilization and release of pro-apoptotic factors in response to intrinsic as well as extrinsic activation [28]. UL36 and UL37 thus act in tandem to suppress apoptosis in CMV-infected cells. Interestingly, UL37 has been previously implicated in controlling species specific cell tropism between human and mouse CMV as insertion of HCMV UL37 enables MCMV to grow in human cells [6]. Moreover, recent evidence indicates that microRNAs expressed by HCMV can exert anti-apoptotic effects [54], and other mechanisms of CMV apoptosis suppression are currently being explored [55–59]. Thus, RM-specific compensatory mechanism might explain the species-specific dependence on UL36 for infection of CM but not RM.

In contrast to UL36, which acts post-entry, the UL128 and UL130 proteins of HCMV and RhCMV represent subunits of the gH/gL/UL128/UL130/UL131A pentameric complex in the viral envelope that facilitates entry into non-fibroblast cells such as endothelial, epithelial, or myeloid cells [26,60]. In both HCMV and RhCMV, repeated passaging in fibroblasts results in selection of pentamer-mutants [25,34]. In the case of RhCMV 68–1, an inversion and loss of genetic information within the ULb' region resulted in deletion of both UL128 and UL130 [25]. RhCMV 68–1 is thus much more limited in the number of different cell types it can infect efficiently *in vitro* and *in vivo* [5,26]. However, insertion of the UL128 and UL130 genes from RhCMV strain 180.92 into RhCMV 68–1 to create RhCMV 68–1.2 fully restored efficient infection of endothelial and epithelial cells [5]. It is thus likely that repair of the pentameric complex in RhCMV 68–1.2 enabled the virus to broadly enter multiple cell types. Since cell types differ with respect to their susceptibility to apoptosis signals [61] this broadening of cell tropism might thus enable infection of CM even in the absence of a functional UL36 protein.

Our finding that both RhCMV 68–1 UL36R and RhCMV 68–1.2 can super-infect CyCMV-infected MCM enables the use of this unique nonhuman primate to determine the contribution of host immunogenetics versus strain-specific CMV mechanisms for induction of unconventional MHC-E and MHC-II restricted CD8+ T cells and remarkable protection from SIV replication previously observed in RhCMV/SIV-vaccinated RM [13]. MCM are a particularly attractive group of CM due to a significant population bottleneck 400 years ago, resulting in highly limited immunogenetics [23,36,62]. Consequently, MCM provide an exceedingly simplified genetic background on which to test various CMV vaccine vectors. Indian rhesus macaques exhibit remarkably complex MHC genetics with each animal expressing up to twenty MHC-I molecules [17], thereby complicating the study of these SIV-protective, RhCMV-induced CD8+ T cell responses. In contrast, MCM have only seven, completely described MHC haplotypes [23,36,62]. Therefore, MCM are the ideal nonhuman primate model in which to study the contribution of immunogenetics to RhCMV-induced protective T cell immunity. In addition, our characterization of CyCMV genomes isolated from MCM will facilitate the molecular cloning of CyCMV, thus enabling the comparison of RhCMV and CyCMV-elicited T cell responses within the homologous or heterologous species. Since CM and RM differ with respect to their susceptibility to certain infectious agents, particularly respiratory pathogens like influenza [63], cross-species comparisons will impact CMV-based vaccine research. Interestingly, the primary MCM isolates exhibited minimal diversity, in accordance with the sharp genetic bottleneck that occurred in this population approximately 400 years ago [36]. Thus, our genetic data bridge an important gap in our understanding of similarities and differences between RhCMV and CyCMV strains, open new avenues of research into understanding the requirements for priming unconventional MHC-E and MHC-II restricted CD8+ T cell responses, and further the utility of both RM and CM in future infectious disease research.

## Materials and Methods

### Construction of recombinant RhCMV

The RhCMV 68–1 BAC has been extensively described [11,12]. RhCMV 68–1.2 has been previously described and was kindly provided by Dr. Thomas Shenk [5]. The RhCMV 68-1/gag, RhCMV 68-1/env, and RhCMV 68–1.2/gag vectors as well as all viruses carrying deletions in the US6 family of T cell evasion genes used in our T cell activation assay have been previously described [15,41,49]. RhCMV 68–1.2/pol was constructed using the same homologous recombination technique previously published for the RhCMV 68-1/pol vector [13,64]. Briefly, an expression cassette containing the 5’-region of the SIVmac239 pol protein with an EFIα promotor and a kanamycin (Kan) resistance cassette were amplified from a pORI-6K-F5 plasmid (primer binding site forward: 5’-ACTTAACGGCTGACATG-3’, primer binding site reverse: 5’-AGCTTAGTACGTTAAAC-3’). The primers used had a 50bp homology to the target region in RhCMV 68–1.2 between Rh213 and Rh214, exactly the same location previously described by us for insertion of other SIV transgenes (forward primer: 5’-CTGGGTAGTCAACATGGGCATACGAAACTTGCCCGAATAGATGCTCTCAC-3’, reverse primer: 5’-CTTTTTGGCCAGCGGGTTGGATGATTTCGCGCGTCATGGACTGCTTCACT-3’) [49]. Since homologous recombination was carried out in E.coli strain SW105, the Kan selection marked was removed by heat shock activating the expression of a Flp recombinase. The virus was reconstituted in primary rhesus fibroblasts and transgene expression confirmed by immunoblot. Rh157.5 (UL128) and Rh157.4 (UL130) deletions were based on the previously described RhCMV 68–1.2/gag vector. Primers with 50bp homology arm to regions upstream and downstream of the targeted ORFs (forward primer UL128: 5’-GTCATGATATAGTTCCGCCTGGCTGTTTAGGCGGCATCCTTCCGGCTAAT-3’, reverse primer UL128: 5’-ATTTTTCGATAAAAAAATCACAGCAAACATACTGGTTTTACACACTTTAT-3’) (forward primer UL130: 5’-AAAACTATAATCAACAACTCTATACCTTTGTTTTGCTGATGCTATTGCGT-3’, reverse primer UL130: 5’-ATTAGCCGGAAGGATGCCGCCTAAACAGCCAGGCGGAACTATATCATGAC-3’) were designed with primer binding sites to pORI-6K-F5 (primer binding site forward: 5’-ACTTAACGGCTGACATG-3’, primer binding site reverse: 5’-AGCTTAGTACGTTAAAC-3’) and a Kan resistance cassette flanked by F5-FRT sites was amplified and inserted in place of UL128 or UL130 by homologous recombination. The selection marker was removed by heat shock to activate the expression of a Flp recombinase. Deletion of the targeted ORF was confirmed by PCR and RT-PCR, and the entire viral genome fully sequenced using Illumina deep sequencing as described below. UL36 was repaired in the 68–1 clone RhCMV/gag using galK recombination. Briefly, the galK gene was inserted between nucleotides 831 and 833 (counted from the ATG start codon) in the UL36 ORF. In a second recombination step, the galK gene was then replaced with a 100mer oligonucleotide that corresponded to the correct WT sequence (AACTTGTCAACTAGTACATAGAGTCTGACTAGGAACTCATTTTTTTCTTTACGGAAGCAACCTAGCACCCCGAGCAATTGATTAA). Successful recombinations were screened by sequencing of PCR products, and once confirmed, each virus was subsequently sequenced by Illumina deep sequencing as described below. To generate the RhCMV 68–1.2/pol ΔRh61/Rh60 (ΔUL36) mutant, the UL36 ORF was replaced by the SIV pol transgene. The same pORI-6K-F5 plasmid containing the SIVmac239 pol protein described above was used as a template, but since the endogenous UL36 promoter was used to drive transgene expression, the PCR product did not contain the EFIα promotor. The primers used to amplify the SIV pol protein had a 50 bp homology to the upstream and downstream region of UL36 (forward primer 5’-TTGTATATATTGTCGTTATGTGATTTATTGCTACACATCAAATAAACATG-3’, reverse primer 5’-TCAGTGAACTCAACGTGGTTCGTCAACAAACATAACCTCAGCTTTGTCGT-3’) and the following primer binding sites for pORI-6K-F5 (primer binding site forward: 5’- GTATGTTGTGTGGAATTGTGAG-3’, primer binding site reverse: 5’-ACCATGCGGGAGGCGTT -3’). After successful recombination, the Kan selection marker was removed as discussed above and transgene expression confirmed by immunoblots of cells infected with the reconstituted virus. To ensure the correct viral genome sequence, viral DNA was analyzed by Illumina deep sequencing as described below.

### Isolation of the novel cytomegaloviruses from RM and MCM

Virus isolation was performed as previously described [65]. Briefly, urine was obtained from late stage SIVmac239-infected rhesus macaques or healthy MCM upon arrival at ONPRC through cystocentesis or following euthanasia. To clarify the urine from cells, debris, and contaminants it was first centrifuged at 2,000 x g for 10 minutes at 4°C and then filtered through a 0.45 μm filter (Millipore) to clear the urine from any bacterial or fungal contamination. Subsequently, primary rhesus fibroblasts were spin-inoculated at 700 x g for 30 minutes at 25°C in 6 well plates by adding 500μl –1,000μl of clarified urine per well. Two hours later the urine was washed off the cells with PBS and new DMEM was added. Cells were kept in culture for up to 1 month or until CPE was observed, after which time the cells were harvested and used to infect new primary rhesus fibroblasts to generate a viral stock for further examination. If virus progress was slow, cells were trypsinized and re-seeded to help the infection spread through the entire monolayer.

### Next generation sequencing of viral DNA

To generate purified viral DNA for next-generation-sequencing, we used a modified Hirt extraction protocol originally designed for the extraction of Polyoma virus DNA from mouse cells [66]. Three T-175 flasks of primary rhesus fibroblasts were infected with our novel CMV isolates at an MOI of 0.1 and the viral supernatants were harvested at full CPE after about 7–10 days. Cellular contaminants and residual cells were removed by centrifugation initially at 2,000 x g for 10 minutes at 4°C and subsequently at 7,500 x g for 15 minutes. The virus was pelleted from the clarified medium by overlaying a sorbitol cushion (20% D-sorbitol, 50 mM Tris [pH 7.4], 1 mM MgCl_2_) and centrifuging at 64,000 x g for 1 hour at 4°C in a Beckman SW28 rotor. The generated pellet was resuspended in 500μl 10.1 TE Buffer (10mM Tris, pH 8.0; 0.1mM EDTA, pH 8.0) and 500 μl 2x lysis buffer (20mM Tris-Cl, pH 8.0; 50mM EDTA, pH8.0; 200mM NaCl; 1.2% w/v SDS) was added. Finally, to digest the purified virions, 250μg Proteinase K was added and the solution was incubated for 2h at 37°C. The viral DNA was phenol/chloroform extracted twice and precipitated with absolute ethanol at −80°C overnight. The DNA was pelleted for 20 minutes at 13,200 x g at 4°C, washed once with 70% ethanol, and subsequently resuspended in water. DNA concentrations were determined using a ND-1000 Spectrophotometer (NanoDrop Technologies, Inc.). Illumina sequencing libraries were generated as previously described [11]. Briefly, DNA was fragmented using an S2 Sonicator and was then converted to libraries using the standard TruSeq protocol. Libraries were examined on a Bioanalyzer (Agilent) and the concentration was determined using real time PCR and SYBR Green fluorescence. Next generation sequencing was performed using a MiSeq Next-Generation Sequencing System (Illumina). Libraries were loaded into a MiSeq reagent cartridge at a concentration of 9 pM and single read sequencing was performed for 300 cycles with 6 additional cycles of index reads. The resulting data was imported into Geneious 8.1.4. and the sequencing reads were trimmed of all regions exceeding the error probability limit of 0.1% to minimize sequencing errors. All reads with a total length of less than 50 bp after quality control were eliminated from further analysis to increase the likelihood of specific alignments during de novo and reference guided assemblies. Genomes of our viral isolates were first *de novo* assembled using the processed sequencing data, and subsequently all reads were aligned to the generated consensus sequence in a reference guided assembly to examine potential SNPs and ensure accuracy. SNPs that showed a frequency of more than 50% in a location with a depth of at least 10% of the mean depth were considered a true change of the consensus sequence, and hence changes to the consensus sequences were applied and referenced guided assemblies were repeated until no SNP showed a frequency of 50% or more.

### Phylogenetic and statistical analysis

Full length nucleotide alignments, amino acid alignments and neighbor-joining phylogenetic trees were generated using ClustalW2 [67]. Sliding window analyses and calculations of nucleotide diversity were performed using Variscan, version 2.0.3 [68]. The sliding window analyses used 100bp windows, incrementing every 50bp. All sequence data were visualized using Geneious, which was also used to generate images of alignments (Geneious version 9.1, Biomatters Inc.).

### Ethics statement

Urine and fibroblast samples were obtained from Indian rhesus macaques (*Macaca mulatta*) housed at the Oregon National Primate Research Center (ONPRC) for other ongoing, unrelated studies previously described [15,41,49]. A total of 12 male and 4 female MCM (*Macaca fascicularis)* originally from Mauritius and housed at the ONPRC were utilized for *in vivo* infection studies under the approval of Oregon Health and Science University (OHSU) Institutional Animal Care and Use Committee (IACUC) Protocol 0967. Primary MCM fibroblasts were a kind gift from Dr. Ole Isacson. All macaques in this study were managed according to the ONPRC animal husbandry program, which aims at providing consistent and excellent care to nonhuman primates. This program is based on the laws, regulations, and guidelines set forth by the United States Department of Agriculture (e.g., the Animal Welfare Act and its regulations, and the Animal Care Policy Manual), Institute for Laboratory Animal Research (e.g., Guide for the Care and Use of Laboratory Animals, 8^th^ edition), Public Health Service, National Research Council, Centers for Disease Control, the Weatherall Report titled “The use of nonhuman primates in research”, and the Association for Assessment and Accreditation of Laboratory Animal Care (AAALAC) International. The nutritional plan utilized by the ONPRC is based on National Research Council recommendations and supplemented with a variety of fruits, vegetables, and other edible objects as part of the environmental enrichment program established by the Behavioral Management Unit. Paired/grouped animals exhibiting incompatible behaviors were reported to the Behavioral Management staff and managed accordingly. MCM were challenged subcutaneously with varying doses of RhCMV/SIV vectors in the upper arm or upper leg. In cases where a single MCM was challenged with multiple RhCMV/SIV vectors, each vector was administered at a distinct anatomic location (arms and legs). All efforts were made to minimize suffering through the use of minimally invasive procedures, anesthetics, and analgesics when appropriate. Animals were painlessly euthanized with sodium pentobarbital and euthanasia was assured by exsanguination and bilateral pneumothorax, consistent with the recommendations of the American Veterinary Medical Guidelines on Euthanasia (2013).

### CMV co-cultures and western blotting

Telomerized rhesus fibroblasts were spin-inoculated with centrifuged filter-sterilized (0.4mm) urine from both RM and MCM at 1,600 x g for 1 hour at 4°C. Following 30 days of co-culture, we prepared cell lysates and assessed RhCMV-SIV vector replication on the basis of expression of SIV antigen by western immunoblotting. The α-HCMV TRS1 mouse monoclonal antibody that cross-reacts with RhCMV as well as CyCMV in immunoblots was a kind gift from Dr. Adam Geballe (University of Washington, Seattle, WA). The α-GAPDH antibody was acquired from Santa Cruz Biotechnology (sc-51906). The α-RhCMV IE1 (2A1.2), α-RhCMV IE2 (11A5.2) and α-RhCMV glycoprotein (6H7.3) antibodies were raised in house by the VGTI Monoclonal Core. Similarly, mouse polyclonal antiserum against RhCMV pp71 was generated by the VGTI Monoclonal Core by immunizing mice with a plasmid encoding for the RhCMV 68–1 pp71 protein.

### Multi-step growth curves of RhCMV on primary RM and MCM fibroblasts

Primary RM or MCM fibroblasts, and telomerized rhesus fibroblasts [69], were maintained in Dulbecco’s modified Eagle’s medium with 10% fetal bovine serum and penicillin/streptomycin (DMEM) at 37°C in humidified air with 5% CO_2_. We infected primary RM or MCM fibroblasts with RhCMV 68–1 or RhCMV 68–1.2 at an MOI of 0.1 (MOI was calculated on titrations of the RhCMV stocks using primary RM fibroblasts) and collected supernatant on days 1, 3, 5, and 7. Limiting dilution plaque assay was used to assess viral titers. As such the dilutions of the viral supernatants were added to immortalized RM fibroblasts and overlaid DMEM-10 containing carboxymethyl-cellulose. Plaque formation was assessed 7 days later by fixing the plates with formalin followed by staining with methylene-blue dye.

### CD8+ T cell IFN-γ ELISpot

SIVnef-specific CD8+ T cell lines were generated from SIV-infected RM and MCM as previously described [70]. MHC-matched, primary RM and MCM fibroblasts were infected 48 hours prior to assay set-up with various RhCMV clones expressing a fusion protein of SIV rev, tat, and nef (RhCMV/rtn) at an MOI of 1. These targets were assessed for infection by FACS analysis using an anti-IE2 antibody, and the number of infected targets was normalized across each condition. SIVnef-specific CD8+ T cell lines were mixed with RhCMV/rtn-infected or Nef peptide-pulsed targets at an E:T ratio of 10:1 overnight in Monkey IFN-γ ELISpot plates (Mabtech, Cincinnati, OH). Plates were processed according to the manufacturer’s recommendation and read using an AID plate reader. Data were normalized to the CD8+ T cell IFN-γ release measured after Nef peptide-pulsed fibroblast stimulation.

### Analysis of T cell responses against RhCMV/SIV vectors

SIV-specific CD4+ and CD8+ T cell responses were measured in mononuclear cell preparations from blood and BAL fluid by flow cytometric intracellular cytokine analysis, as previously described [49]. Briefly, sequential 15-mer peptides (overlapping by 11 amino acids) comprising the SIVmac239 Gag, Pol, or Env proteins were used in the presence of co-stimulatory CD28 and CD49d monoclonal antibodies (BD Biosciences). Cells were incubated with antigen and co-stimulatory molecules alone for 1 hour, followed by addition of Brefeldin A (Sigma-Aldrich) for an additional 8 hours. Co-stimulation without antigen served as a background control. Cells were then stained with fluorochrome-conjugated monoclonal antibodies, flow cytometric data were collected on a LSR II (BD Biosciences), and data were analyzed using FlowJo software version 10.0.8 (Tree Star). Response frequencies (IFNg+/TNFa+) were first determined in the overall CD4+ and CD8+ population and then memory corrected (with memory T cell subset populations delineated on the basis of CD28 and CD95 expression).

### Nucleotide and protein sequences generated and used in this study

The sequence information for the novel NHP CMV isolates described in this study, RhCMV 19262 (KX689267), RhCMV 19936 (KX689268), RhCMV 24514 (KX689269), CyCMV 31906 (KX689263), CyCMV 31907 (KX689264), CyCMV 31908 (KX689265) and CyCMV 31909 (KX689266) have been submitted to GenBank. During our phylogentic analysis, the following DNA and amino acid sequences found in GenBank were used: GPCMV 22122 (KC503762, AGE11533), MCMV Smith (GU305914, P27172), AoHV-1 S34E (FJ483970, AEV80760), SaHV-4 SqSHV (FJ483967, AEV80915), CyCMV Ottawa (JN227533, AEQ32165), CyCMV Mauritius (KP796148, AKT72642), RhCMV 68–1 (JQ795930, AFL03576), RhCMV 180.92 (DQ120516, AAZ80589), SCMV Colburn (FJ483969, AEV80601), SCMV GR2715 (FJ483968, AEV80365), DrCMV OCOM6-2 (KR297253, AKI29779), BaCMV OCOM4-37 (AC090446), BaCMV OCOM4-52 (KR351281, AKG51610.1), CCMV Heberling (AF480884, AAM00704), PpygCMV 1 (AAM89279), HCMV TR (KF021605, AGL96654) and GgorCMV 2.1 (ADY62519).

## Supporting Information

S1 Table List of open reading frames (ORFs) truncated, elongated, or deleted in each CMV isolate investigated for these studies. (PDF)
