# Supplementary material for: Cross-Species Rhesus Cytomegalovirus Infection of Cynomolgus Macaques
Source: PLoS Pathog. 2016 Nov 9;12(11):e1006014. doi: 10.1371/journal.ppat.1006014 (PMC5102353; doi:10.1371/journal.ppat.1006014)
Supplement: S1 Table — (PDF) [file ppat.1006014.s001.pdf]

Supplemental table 1.

Isolate	OFRs truncated	ORFs elongated	ORFs deleted	Total ORFs affected
RhCMV 68-1	Rh08 (RL11D), Rh61/Rh60 (UL36), Rh152/Rh151 (UL119/UL118), Rh161 (UL146G), Rh197 (US14D)	Rh13.1 (RL11G, RL13)	Rh157.5 (UL128), Rh157.4 (UL130), Rh158.2 (UL146B), Rh158.3 (UL146C), Rh161.1 (UL146E)	11
RhCMV 68-1.2	Rh08 (RL11D), Rh152/Rh151 (UL119/UL118), Rh157.4 (UL130), Rh161 (UL146G), Rh167 (O14), Rh197 (US14D)	Rh13.1 (RL11G, RL13)	Rh158.2 (UL146B), Rh158.3 (UL146C), Rh161.1 (UL146E)	10
RhCMV 68-1 FL	Rh08 (RL11D)	-	-	1
RhCMV 180.92	Rh10 (COX-2), Rh13.1 (RL11G, RL13), Rh21 (RL11K), Rh148 (UL116), Rh157.4 (UL130), Rh220 (US28F)	Rh06 (RL11B), Rh08.1 (RL11E), Rh167 (O14)	Rh159 (UL148), Rh158 (UL147), Rh158.1 (UL146A), Rh158.2 (UL146B), Rh158.3 (UL146C), Rh161.1 (UL146E), Rh161 (UL141G), Rh162 (UL145), Rh163 (UL144), Rh164 (UL141), Rh164.1 (O11), Rh165 (O12), Rh166 (O13)	22
RhCMV 19262	Rh08.1 (RL11E), Rh23 (RL11M), Rh158 (UL147), Rh158.1 (UL146A), Rh158.3 (UL146C), Rh161 (UL146G)	Rh13.1 (RL11G, RL13)	-	7

RhCMV 19936	Rh07 (RL11C)	Rh06 (RL11B), Rh08.1 (RL11E)	-	3
RhCMV 24514	Rh08 (RL11D), Rh13.1 (RL11G, RL13), Rh161 (UL146G)	-	-	3
CyCMV Ottawa	Cy08 (RL11D), Cy112 (UL83B)	Cy06 (RL11B)	Cy08.1 (RL11E), Cy10 (COX-2), Cy12 (RL11F), Cy13.1 (RL11G, RL13), Cy22 (RL11L), Cy179 (O23)	9
CyCMV Mauritius	Cy08 (RL11D)	Cy07 (RL11C)	Cy08.1 (RL11E), Cy10 (COX-2), Cy12 (RL11F), Cy13.1 (RL11G, RL13), Cy17 (RL11H), Cy19 (RL11I), Cy22 (RL11L)	9
CyCMV 31906	Cy08 (RL11D), Cy13.1 (RL11G, RL13)	Cy07 (RL11C)	Cy08.1 (RL11E), Cy22 (RL11L)	5
CyCMV 31907	Cy08 (RL11D), Cy20 (RL11J)	Cy07 (RL11C)	Cy08.1 (RL11E), Cy22 (RL11L)	5
CyCMV 31908	Cy08 (RL11D), Cy13.1 (RL11G, RL13)	Cy06 (RL11B), Cy07 (RL11C)	Cy08.1 (RL11E), Cy22 (RL11L)	6
CyCMV 31909	Cy08 (RL11D), Cy13.1 (RL11G, RL13)	Cy06 (RL11B), Cy07 (RL11C)	Cy08.1 (RL11E), Cy22 (RL11L)	6
